# Research at the Seat of War

**Published:** 1916-01-29

**Authors:** 


### RESEARCH AT THE SEAT OF WAR.

### The Combating of Wound Infections.

According to a recent announcement, a distinguished expert in tropical diseases, Dr. Aylmer May, who has lately been Principal Medical Officer of the Forces in Northern Rhodesia, has been selected by the War Office to undertake research on the Western Front in connection with wound infections. It is permissible to speculate whether and to what extent the work of the Research Hospital at Compiègne, controlled by Dr. Alexis Carrel of the Rockefeller Institute in New York, has stimulated the authorities of our Army Medical Service to do likewise; and why they have waited so long. At least, it is something to the good that serious attention is to be given to this question in our Army. The Compiègne researches, so far as they have been made public, have not disclosed any extremely brilliant and epoch-making discoveries; though some decidedly valuable work has been done which has already favourably influenced therapeutic practice. One of the latest papers, for instance, published from Dr. Carrel's hospital (on "gas gangrene") has explained, from a scientific standpoint, the *rationale* of that treatment of deep infected wounds which has already been found to answer best in actual practice; and may serve to diminish the fear of that deadly complication by showing how best to attack and defeat it. A vast amount of painstaking research into very many different antiseptics has also been put on record; and the lessons thus learned are proving, by all that we hear, useful and fruitful of good results.

There are, however, certain drawbacks to be anticipated from the appointment of an official research worker, excellent though the recognition of scientific research is in most ways. An impression has got about that individual officers of the Royal Army Medical Corps who have endeavoured to carry on research during those intervals of hospital slackness of which a good deal has been heard (corresponding to periods of comparative inactivity at the Front), have received no encouragement at all from highly-placed authorities of the Army Medical Service, and have even been so severely snubbed that they have preferred to resign their commissions. Not so very long ago a most distinguished scientist, once a professor of the Royal Army Medical Corps, put forward in a book the suggestion that the individual medical officer's discretion in respect of surgical treatment should be taken from him, and that uniformity of practice should be enforced as a disciplinary measure from above. The same distinguished man, whose genius and fertility of ideas are undisputed, had not long before advocated a novel system of treatment for wound infections for which the most wonderful results were claimed. After a pretty good trial the verdict of the clinicians was not nearly so definitely laudatory as the inventor had supposed it would be, and the whole matter afforded a very good object-lessson in the disadvantages of restriction of the discretion of the individual surgeon how he should treat his patients. The conceivable drawbacks to the appointment of an official researcher in wound infections are mainly two: one, that the prestige derived from his position may clothe his results with too much authority, especially if they are unchecked by private research among the rank and file of the pathologists; and the other, that he may prove to be too pliable in the hands of the high authorities, who may possibly be wedded to hypotheses and theories which they are unwilling to see controverted.

In short, the notes of warning which should, in our judgment, be sounded are briefly as follows. Not too much should be expected immediately from this appointment; for Carrel and a band of fellow-workers have been working at the same problems for a year and a half without reaching a final solution. There should be no discouragement of independent research outside the laboratory of the newly-appointed official researcher. The latter should be rendered as far as possible independent of the hierarchy of the Army Medical Service, in the senses that he should be quite free from interference or influence by senior officers; and that if his results conflict with their views or opinions, he should be independent of them for recognition and reward for his services. This condition may, no doubt, be difficult to secure; and much must necessarily depend on the character and scientific temper of the newly-appointed officer himself. We sincerely trust that his labours will result in great and lasting additions both to scientific knowledge and to practical therapeutics.

